# Impacts of cognitive impairment for different levels and causes of traumatic brain injury, and education status in TBI patients

**DOI:** 10.1590/1980-57642018dn12-040012

**Published:** 2018

**Authors:** Minoo Sharbafshaaer

**Affiliations:** 1 Young Researchers and Elite Club, Zahedan Branch, Islamic Azad University, Zahedan, Iran.

**Keywords:** cognitive impairment, traumatic brain injury (TBI), education status, TBI patients, comprometimento cognitivo, traumatismo cranioencefálico (TCE), grau de instrução, pacientes lesionados

## Abstract

Traumatic brain injury (TBI) is one of main causes of death and disability among many young and old populations in different countries.

**Objective:**

The aim of this study were to consider and predict the cognitive impairments according to different levels and causes of TBI, and education status.

**Methods:**

The study was performed using the Mini-Mental State Examination (MMSE) to estimate cognitive impairment in patients at a trauma center in Zahedan city. Individuals were considered eligible if 18 years of age or older. This investigation assessed a subset of patients from a 6-month pilot study.

**Results:**

The study participants comprised 66% males and 34% females. Patient mean age was 32.5 years and SD was 12.924 years. One-way analysis of variance between groups indicated cognitive impairment related to different levels and causes of TBI, and education status in patients. There was a significant difference in the dimensions of cognitive impairments for different levels and causes of TBI, and education status. A regression test showed that levels of traumatic brain injury (b=.615, p=.001) and education status (b=.426, p=.001) predicted cognitive impairment.

**Conclusion:**

Different levels of TBI and education status were useful for predicting cognitive impairment in patients. Severe TBI and no education were associated with worse cognitive performance and higher disability. These data are essential in terms of helping patients understand their needs. Therefore, the factors identified can help plan effective rehabilitation programs.

## RESUMO

A lesão cerebral traumática (TCE) é uma das principais causas de morte e incapacidade em muitos jovens e idosos em diferentes países.

**Objetivo:**

O objetivo deste estudo foi considerar e prever os prejuízos cognitivos para os diferentes níveis e causas do TCE e status de educação.

**Métodos:**

O estudo foi feito usando o Mini-Exame do Estado Mental (MMSE) para estimar o comprometimento cognitivo em pacientes dirigido a um centro de trauma na cidade de Zahedan. Os indivíduos foram considerados elegíveis se tivessem 18 anos de idade ou mais. Esta investigação avaliou um subconjunto de pacientes de um estudo piloto de 6 meses.

**Resultados:**

Os participantes do estudo foram 66% do sexo masculino e 34% do sexo feminino. A média de idade dos pacientes foi de 32,5 anos e DP foi de 12,924 anos. A análise de variância unidirecional entre grupos indicou comprometimento cognitivo relacionado a diferentes níveis e causas de TCE e status de educação em pacientes. Houve uma diferença significativa nas dimensões de deficiências cognitivas para os diferentes níveis e causas de TCE e status de educação. O teste de regressão mostrou que níveis de lesão cerebral traumática (b=0,615, p=0,001) e o status de educação (b=0,426, p=0,001) predizem o comprometimento cognitivo.

**Conclusão:**

Os diferentes níveis de TCE e estados de educação foram uteis para prever o comprometimento cognitivo em pacientes. TCE grave e sem educação foram relacionadas a piora do desempenho cognitivo e maior incapacidade. Esses dados são essenciais para ajudar os pacientes a entender o que realmente precisam, portanto, os fatores identificados podem contribuir no planejamento de programas de reabilitação efetivos.

Traumatic brain injury (TBI) is the most common cause of death and disability among young and old persons in many countries, including the USA and Canada,^1^ where TBI results in chronic neurological, cognitive, and behavioral impairments.^2^ It can, therefore, be one of the most challenging and rewarding aspects of clinical neurocritical care.^3^ Traumatic brain injury patients are at high risk for impairments in pragmatic language and social communication in general.^4^ Post-traumatic brain-injured patients, after coming out of coma, specifically report behavioural problems and cognitive impairments.^5^

Cognitive impairment is a common consequence of traumatic brain injury and a substantial source of disability, across all levels of TBI severity, where attention, processing speed, episodic memory, and executive function are the most commonly affected dimensions.^6^ Cognitive impairment is a common outcome at each levels and causes of TBI in patients. In one study, during follow-up after trauma, all patients underwent neurological examination including the Mini-Mental State Examination (MMSE) and Glasgow outcome scale (GOS).^7^ Another study showed that moderate-to-severe TBI patients reported cognitive deficits including memory, language, executive functions, attention and information processing speed impairments.^8^ TBI patients are at risk of subjective memory impairment, which was found to be significantly greater among TBI patients with loss of consciousness, where post-TBI cognitive impairment primarily affects executive function and processing speed.^9^

However, there was a statistically significant increase in the composite cognitive score and decrease in functional connectivity in the right inferior frontal gyrus, with changes in the brain-behavior in Traumatic brain injury patients.^10^ White matter disruption after brain injury indicates cognitive impairment, where white matter damage was associated with particular patterns of cognitive impairment.^11^ Cognitive function was associated with outcomes, and therefore screening of cognitive function could be of importance in a clinical setting.^12^

Furthermore, severity levels of traumatic brain injury determine the degree of cognitive impairment. Levels of traumatic brain injury include severe, moderate or mild, and can have different outcomes in terms of cognitive impairment. A study reported that moderate-to-severe traumatic brain injury can cause varying degrees of cognitive control deficits, with a positive relationship to injury severity correlated with self-reported cognitive control problems in everyday-life situations.^13^ Traumatic brain injury is a risk factor for cognitive decline in mild cognitive impairment among older adult patients.^14^ Patients with mild traumatic brain injury had worse neurocognitive function, higher overall symptom severity and higher total number of symptoms. There is a cognitive deficit and symptom burden in patients with TBI.^15^

Traumatic brain injury is usually caused by a strike or other traumatic injury to the head or body.^16^ The most common causes of TBI are falls and vehicle accidents, followed by acts of violence and other reasons, such as fight injuries. Also, different causes of TBI are associated with different cognitive impairments. For example, different cognitive impairments are reported in car accidents, head trauma and falls.

Education plays an important role in cognitive impairment following TBI. The literature shows that education appears to affect verbal and nonverbal task performance in mild cognitive impairment patients. While higher educated patients may be more acquainted with the tasks, slower deterioration in consecutive follow-up examinations could be explained by the cognitive reserve theory.^17^

Comprehensive neuropsychological assessments in each cases of TBI are important to identify impaired and preserved functions, thereby allowing adequate management, including rehabilitation programs, for each cases.^18^ Therefore, the aims of this study were to assess and predict cognitive impairments for different levels and causes of TBI, and education status in traumatic brain injury patients. These three factors can help plan effective rehabilitation programs.

## METHODS

### Participants and Procedures for data collection

This study examined a subset of patients from a pilot study with sample selection using the random method. The investigation covered a 6-month period from March 2017 to October 2017. The study was conducted at a level one trauma centre in Zahedan city. The patients were receiving medical care. Subjects were considered eligible if they were 18 years of age or older and had sustained one of five kinds of traumatic brain injuries within 24 hours of presentation to the Emergency Department. Children and patients with previous orthopaedic conditions were excluded.

Data regarding the levels of severe Brain Injury, moderate and mild Traumatic Brain Injury and causes of the injury, including car accident, car accident multiple trauma, head trauma, head trauma multiple trauma, and fall were also collected within 2 months of patient discharge. Generally, the study was completed within 6 months.

### Demographic questionnaire

At the beginning research, considering degrees of Traumatic Brain Injury by Glasgow Coma Score (GCS) test has been done, 9-12 score is moderate TBI, and GCS of 3-8 is severe TBI. Patients were enrolled prospectively after providing written informed consent and completed the Mini-Mental State Examination (MMSE) questions. This is a well-validated scale for cognitive impairment in Iranian adults. Cognitive tests provide valuable information for understanding the mechanism of cognitive impairment after traumatic brain injury and for the management of TBI patients.^19^ Investigation indicated that MMSE serves as a similar predictor to the Disability Rating Scale in TBI patients.^20^ The validity of the Persian MMSE in recognizing normal versus abnormal cognition cut-off score for Persians has been demonstrated, with a sensitivity and specificity of 98% and 100%, respectively. The test usually takes about ten minutes to complete. The MMSE evaluates a range of cognitive domains, including orientation, memory, language, attention and calculation, and the ability to follow simple verbal and written commands.^21^ The MMSE is usually used as a brief test in clinics and as a research instrument for assessing cognitive impairment and dementia. The MMSE serves as a similar predictor to the Disability Rating Scale at discharge.^22^ Scores of 25-30 out of 30 are considered normal; the National Institute for Health and Care Excellence (NICE) classifies 21-24 as mild, 10-20 as moderate and<10 as severe impairment.^23^

### Data analysis

Data were analyzed with SPSS, version 21.0 for Windows. Data were reported as mean frequencies. Parametric tests were used, thus, one-way ANOVA test assessed each dimensions of cognitive impairment for the different levels and causes of TBI with education status separately. A regression test was used to predict cognitive impairment according to the different levels and causes of TBI with education. Variables were considered significant (*p<0.05*) with a cut-off for rejection of variables from the model of *p=0.10.*

### Ethical aspects

This study was approved by the Zahedan University of Medical Sciences. Participants were asked to sign the Free and Informed Consent Form, after due clarification concerning the study and before data collection.

## RESULTS

The pilot study included 50 patients, comprising 66% males and 34% females. The mean age of the patients was 32.5 years (range 18-66 years) and SD was (12.924 years, with a 95% confidence interval. The following mechanisms of injury were reported when the patients presented to the Emergency Department (ED): Severe Brain Injury (66%); Moderate TBI (50%); and Mild TBI (40%).

In terms of trauma causes reported, car accident (26%); car accident multiple trauma (38%); head trauma (24%); head trauma multiple trauma (25%); and falls (10%). The data for education status showed No education (24%); School education (62%); University education (14%).

One-way between groups analysis of variance was conducted to assess the impact of different levels and causes of TBI, and education status, as measured by the dimensions of cognitive impairment. There were statistically significant differences in the different levels and causes of traumatic brain injury with education statuses, as estimated by dimensions of cognitive impairment (MMSE). There was a statistically significant difference at the *p<*.05 level for the three levels of TBI assessed (Severe, Moderate, and Mild Traumatic Brain Injury) and all cognitive impairment dimensions were associated with levels of TBI, except Language *F* (2, 47)=2.01, *p*=.14 which showed no statistically significant difference for TBI level. In addition, there was a statistically significant difference, at the *p-value<*.05, for all causes of TBI for all dimensions of cognitive impairment. Education status, divided into three levels (No education, School education, and University education), exhibited a statistically significant difference, at the *p-value<*.05, for dimensions of cognitive impairment, but Registration *F* (2, 47)=1.47, *p* =.23 and Recall *F* (2, 47)=1.63, *p* =.20 displayed no statistically significant difference in cognitive impairment with education status (Table 1).

**Table 1:** Impacts of levels and causes of traumatic brain injury and education on dimensions of cognitive impairment.

Cognitive impairment	Levels(Severe, Moderate, Mild Traumatic Brain Injury)			Causes(CA, CAMT, HT, HTMT, FALL)			Education(No education, school, university education)	
	F	Sig		F	Sig		F	Sig
Orientation	18.74	.001		5.18	.002		5.75	.006
Registration	6.62	.003		3.5	.014		1.47	.23
Recall	22.5	.001		4.28	.005		1.63	.20
Attention and calculation	13	.001		10.7	.001		7.46	.002
Language	2.01	.14		3.41	.016		8.91	.001

Standard multiple regression was used to assess the ability of the three measures (TBI levels, causes of TBI, and education) to predict levels of cognitive impairment. Preliminary analyses were conducted to ensure no violation of the assumptions of normality, linearity, multicollinearity and homoscedasticity. TBI levels, causes of TBI, and education explained 7% of the variance in cognitive impairment. Test scores attained were regressed on the different levels of TBI and education status, indicating that these can predict cognitive impairment, which proved highly significant, *R^2^* =.57, *F*(3,46)=24, *p=*.001, 99% CI. The different levels of traumatic brain injury (b=.615, *p*=.001) and education status (b=.426, *p*=.001) demonstrated significant effects on cognitive impairment with a 99% of confidence interval (Table 2).

**Table 2:** Prediction of cognitive impairment by levels and causes of traumatic brain injury and education.

Cognitive impairment	B	Beta	T	Sig
Levels	4.8	.615	6.24	.001
Causes	.073	.017	.175	.862
Education	3.5	.426	4.58	.001

**Figure 1:**
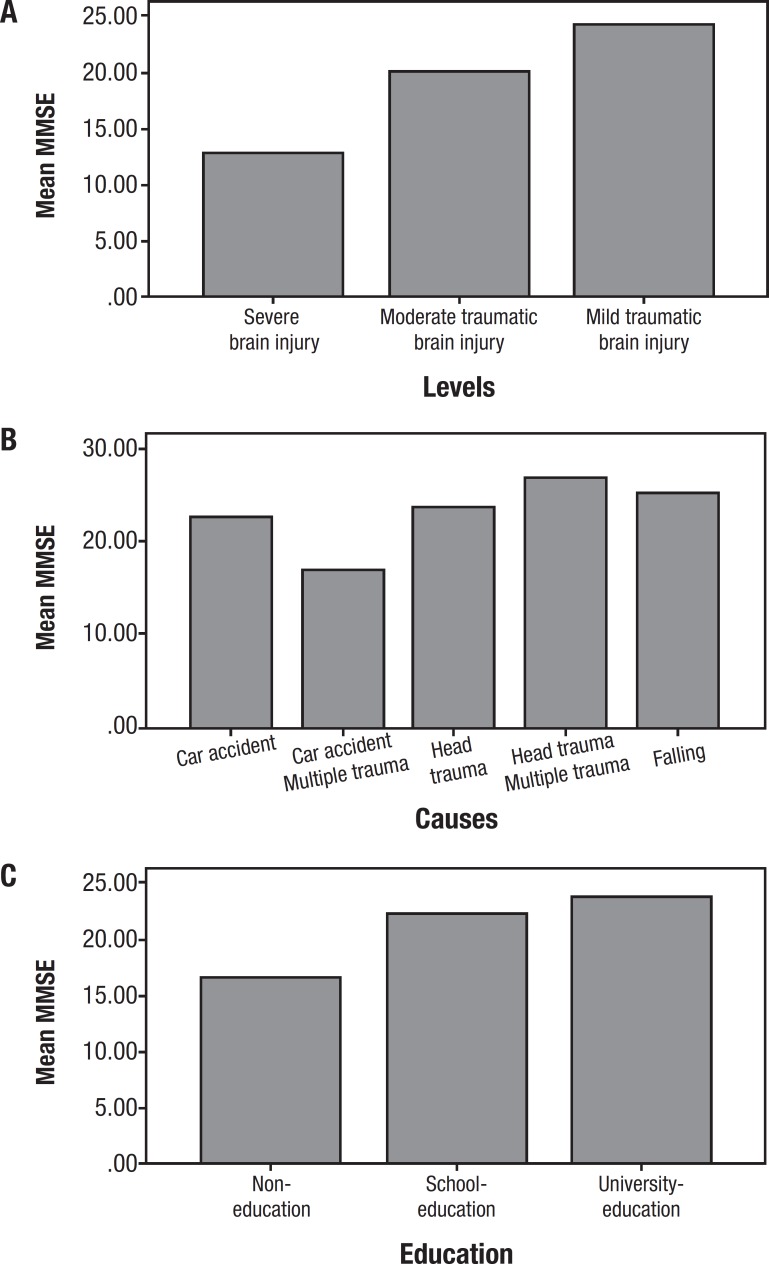
Cognitive impairment (MMSE) according to different levels and causes of TBI and education status

Severe brain injury patients had scores of 10-15, indicating moderate impairment. Moderate and mild TBI cases exhibited mild level of impairment (Figure 1A). Car accident multiple trauma caused moderate level of impairment, whereas Car accident and head trauma caused mild level of impairment. Falls and head trauma multiple trauma showed a normal level of cognitive impairment (Figure 1B). Patients with no education showed a moderate level of impairment, while patients with school and university education scored 20-25, indicating a mild level of cognitive impairment (Figure 1A).

## DISCUSSION

In this study, there was a direct relationship between different levels of TBI and cognitive impairment. Hence, mild-to-severe traumatic brain injury (TBI) led to different degrees of cognitive impairment, which in turn had a negative impact on long-term functional outcomes. Recently published studies on levels of TBI reported that mild traumatic brain injury (mTBI) patients had long-term cognitive impairment.^24^ These patients also had dynamic changes in the thalamus and white matter (WM) as well as cognitive impairment during the follow-up period.^25^ A similar study indicated differences between moderate and severe TBI patients in cognitive deficits, with problems in executive dysfunction, attention and improvement over time.^26^ In contrast, some researchers hold that TBI influences cognition in both moderate and severe cases. Thus, cognitive impairment may be associated with future disability in severe and moderate traumatic brain injury patients.^27^

Causes of traumatic brain injury are an important variable for determining cognitive impairment, indeed different causes of TBI indicated different degrees of cognitive impairment. In particular, car accident multiple trauma, car accident and head trauma indicated high risk of cognitive impairment. Different injuries commonly seen are caused by motor vehicle accidents. Car accidents are associated with serious brain injuries which can lead to numerous cognitive problems and symptoms.^28^ A previous study showed acute head injury is likely and predicts cognitive impairment.^29^ Researchers argued that there is strong evidence that global cognitive impairments are associated with serious fall-related injury, which was also associated with increased risk.^30^

Cognitive impairment was related with education status, which could be predicted by education. In particular, education is strongly associated with levels of cognitive function, but not with rate of cognitive decline. Furthermore, related research has stated that education is correlated with risk of dementia in elderly TBI patients,^31^ although education-cognition relationship are partially explained by intellectual activities.^32^ Another investigation found that higher educated patients performed better on both memory tests at baseline, compared to less educated patients.^33^ In addition, memory and language functions were found to be more resistant to decline in the high-education group.^17^ Evidence indicated that higher education can attenuate the negative effect of TBI on cognitive outcomes.^34^ Educated patient status proved an independent predictor of 1-year disability-free recovery even after traumatic brain injury was adjusted for other prognostic factors.^35^ Mild cognitive impairment patients with higher education/occupation had more elevated regional cerebral metabolic rates for glucose reduction than individuals with low education, and comparable levels of cognitive impairment.^36^

Our study suggests that the different levels and causes of TBI and education status are useful variables for predicting cognitive impairment in patients. Also, TBI affects most in cognitive functions. In fact, different levels (mild-to-severe) and causes of TBI, and education status play potential compensatory role in indicating severity of cognitive impairment. Another study in moderate-to-severe TBI survivors reported that cognitive deficit is associated with functional changes in the brain.^37^ Based on these arguments, cognitive reserve could be a factor driving neural adaptation during recovery from TBI.^35^ However, education had a considerable effect in determining cognitive impairment in traumatic brain injury. Researchers noted that patients with more education have a greater cognitive reserve, or the brain’s ability to maintain function in spite of damage. Higher educated patients have been shown to display fewer symptoms of the disease than individuals with less education, and be more likely to fully recover.^38^ A health education program promoting knowledge to increase understanding about TBI via two modes of delivery, classroom and electronic, was evaluated. Both modes appeared equally effective in terms of self-report of change in long-term TBI knowledge outcomes.^39^

Cognitive impairments following TBI are common and vary widely. However, there are different cognitive rehabilitation techniques and combinations, especially pharmacotherapy, that are helpful in addressing various cognitive deficits.^40^ Also, different levels and causes of TBI and education status should be considered when planning future rehabilitation programs.

The present study was rigorously prepared yet there were statistically significant differences for different levels and causes of traumatic brain injury and education status, as estimated by dimensions of cognitive impairment. This indicates that different levels and causes of TBI and education in patients are variables impacting score achieved across all dimensions of cognitive impairment. The results of this study confirm that different levels and causes of TBI and education status play an important role in cognitive deficit. These factors, therefore, should be addressed in rehabilitation programs. In addition, the present study had limitations, where the duration of this study was relatively short (6 months). Thus the long-term impact of the level and cause of TBI and of education on cognitive impairment should be investigated in larger groups of TBI patients.

In conclusion, the different levels and causes of traumatic brain injury, and education status, correlated with dimensions of cognitive impairment, and TBI levels and education status were useful for predicting cognitive impairment in patients. Severe TBI and no education were associated with worse cognitive performance and higher disability. These data are especially important in terms of helping patients understand their needs. Therefore, the factors identified can help plan effective rehabilitation programs.
